# Cardiovascular outcomes of Nephrotic syndrome in childhood (CVONS) study: a protocol for prospective cohort study

**DOI:** 10.1186/s12882-018-0878-5

**Published:** 2018-04-03

**Authors:** S. K. Patnaik, P. Kumar, M. Bamal, S. Patel, M. P. Yadav, V. Kumar, A. Sinha, A. Bagga, M. Kanitkar

**Affiliations:** 1 Department of Pediatrics, Army Hospital Research and Referral , Delhi, India; 2 Division of Pediatric Nephrology and ICMR Center for Advanced Research In Nephrology, Department of Pediatrics, All India Institute of Medical Sciences, New Delhi, India; 3 Armed Forced Medical College, Pune, India

**Keywords:** Nephrotic syndrome, Prediction, Outcome, Endothelial dysfunction, Laser Doppler Flowmetry, Cardiovascular disease, Children, Study protocol

## Abstract

**Background:**

Nephrotic syndrome (NS) is characterized by dyslipidemia which is a well-known risk factor for atherogenesis. Atherosclerosis in childhood is mostly subclinical and endothelial dysfunction is known to precede this. Evidence for screening for endothelial dysfunction and cardiovascular risk factors and early identification of premature onset of atherosclerosis in childhood NS remains tenuous in the absence of well-designed prospective studies addressing cardiovascular comorbidity in NS. The objective of our study is to examine endothelial dysfunction and short-term cardiovascular outcomes in a carefully phenotyped cohort of patients with Nephrotic syndrome as compared to healthy controls.

**Methods:**

In a multi-centric prospective cohort study, 70 Steroid Resistant NS (SRNS), 70 Steroid Sensitive (SSNS) patients along with 70 Healthy Controls are being recruited. After a baseline assessment of functional and structural status of heart (2D Echocardiography), arteries (Carotid Doppler and Intima Media Thickness measurements) and microcirculation [a combination of 2D Echocardiography, Laser Doppler Flowmetry (LDF) and Brachial Artery Flow mediated dilation (FMD) and Nail Fold Capillaroscopy (NFC)], the patients are being investigated for endothelial dysfunction. Venous blood sample (15 ml) is being collected for routine investigations and assay of biochemical endothelial markers through Flow Cytometry. The patients will be followed up at 12 months and 24 months after the recruitment to look for any change from baseline period.

**Discussion:**

This study will able to provide a better understanding of the epidemiology of endothelial dysfunction and associated subclinical cardiovascular co-morbidity in childhood NS. Findings on characterization of prevalence of endothelial dysfunction and subclinical markers may be used to design future randomized controlled trials for evaluating the efficacy of preventive and therapeutic interventions in reducing the incidence of cardiovascular disease.

## Background

Nephrotic syndrome (NS) is a common pediatric renal disorder in India. With an estimated prevalence of 12–16 cases per 100,000 population and annual incidence of ~ 1.5–2 new cases per 100,000 population, at least 150,000 to 200,000 cases exist amongst Indian children and about 10,000 new cases are added every year [1, 2]. A child with NS gets exposed to multiple risk factors well known to be atherogenic and contributory to progression of chronic kidney disease (CKD) amongst adults [3, 4].

Apart from hyperlipidemia, a well-known risk factor for coronary artery disease (CAD) and a defining hallmark of NS, these children also get exposed to other risk factors associated with impaired endothelial function and adverse cardiovascular outcomes like high oxidative stress, frequent infections, sustained proteinuria, hypoalbuminemia, thromboembolism, toxicity of steroids and non-steroidal drugs like calcineurin inhibitors (hyperlipidemia,vasculotoxic and nephrotoxic effects). Many children with steroid resistant NS progress towards CKD which is known to increase cardiovascular mortality 500 to 1000 fold compared to the general population in young adults [5]. While hyperlipidemia is usually intermittent and transient in steroid sensitive nephrotic syndrome (SSNS), dyslipidemia and other abnormalities may be persistent in patients with frequent relapses (FRNS) or steroid resistant (SRNS) cases [6].

Biological plausibility of extra-renal endothelial alterations in NS arises from a longstanding recognition of increased glomerular endothelial permeability due to an ill-defined ‘permeability factor’ of extra renal origin. Mediators like platelet activating factor, VEGF family, adhesion molecules of selectin family and Nitric Oxide (NO) pathway are involved in the recruitment of inflammatory interstitial cells as well as affect endothelial cells with widespread vascular effects [1]. Occurrence of hemostatic abnormalities and generalized edema in NS attest to the wide ranging effects related to vascular endothelial dysfunction.

Very few original research studies have addressed endothelial alterations in pediatric NS. Most of the existing published data has related to uncontrolled cross-sectional studies in small number of adults with preponderance of non-minimal change disease. Broadly 3 approaches have been adopted –.

### Vascular function studies

Impaired endothelial responsiveness to specific vasodilator stimuli as a surrogate marker of cardiovascular risk has been studied in both micro-vessels and conduit arteries. Post-occlusive Flow mediated dilatation (FMD) of conduit vessels like radial/brachial artery is a functional assay of nitric oxide bioavailability. Few cross-sectional studies of FMD in NS till date do show a 2–3 fold impairment related to hyperlipidemia but it remains difficult to assess the applicability owing to variations related to small sample sizes, relapse rates and pharmacotherapy. Endothelial function at microcirculatory level has seldom been evaluated in children [7]. Coronary flow reserve, a index of coronary microvascular and endothelial function measured by 2D-Echo, was significantly impaired in adult NS cases versus controls and was related to proteinuria, serum creatinine, total cholesterol and inflammation [8]. Pediatric echocardiographic data for cardiac endothelial dysfunction remains scanty till date.

### Biochemical markers of endothelial dysfunction

Endothelial dysfunction is mediated by NO and all NO synthetases (NOS) are constitutively expressed in the kidney. Serum cGMP, TxA2 and PGI2 have also been found elevated in children with NS [6]. Human serum albumin increases the generation of reactive oxygen species and increases ADMA generation by endothelial cells in a dose-and-time-dependent manner in rat models of NS [6]. While studies have addressed NO pathways in relation to steroid responsiveness, no study has primarily looked at NO pathways in relation to endothelial dysfunction in children with NS. CD146 is a novel cell adhesion molecule localized at the endothelial junction that increased in physiopathological settings linked to endothelial junctional alteration [9–11]. sCD146 circulates in the plasma of healthy subjects. Modifications of its basal levels could reflect alterations of junctional functions such as vascular permeability. In the only study of CD146 in relation to other markers of endothelial cell injury in patients with NS, amongst 45 CAPD patients, 43 patients with nephrotic syndrome and 25 healthy volunteers, amongst various markers of endothelial cell injury, all markers except selectins were significantly elevated in patients with NS and CAPD when compared to the healthy volunteers.

### Circulating angiogenic cells as markers of endothelial dysfunction

Assessment of circulating endothelial cells (CECs), endothelial progenitor cells (EPCs) and their derivatives like microparticles and sCD146 would form a novel approach towards defining endothelial dysfunction in childhood NS. In FSGS, levels of CECs were noted to be higher during active stage [12]. In asymptomatic adults with definite exposure to cardiovascular risk factors, levels of circulating EPCs were a better predictor of vascular reactivity than conventional risk factors [13].

Akin to the dearth of data on endothelial dysfunction in childhood NS, subclinical cardiovascular comorbidity of childhood NS also remains poorly characterized. Ambulatory BP monitoring reveals a high prevalence of systolic hypertension in association with increased carotid intima media thickness (cIMT) and endothelial dysfunction [14]. Symptomatic as well as asymptomatic cardiac thrombi have been described in case reports in both SSNS and SRNS. In cross sectional studies, 50% of children with NS showed left ventricular hypertrophy which correlated with carotid stiffness and systolic hypertension while increased Left Ventricular Mass index was detectable in 19% cases [8, 14]. A positive correlation of increased cIMT with number of relapses has been observed in 3 out of 4 published cross sectional studies in childhood NS [13, 14]. Effect of relapse frequency was notable as early as 1 year of disease duration and persisted even after completion of steroid therapy 4 years to 15 years ago and was related to lipid levels. Multiple abnormal subclinical CVD markers were present in 16% and independently associated only with higher LDL cholesterol.

Experimental and clinical studies suggest that increased cholesterol levels, hypoalbuminemia and reduced oncotic pressure induce endothelial cell dysfunction. Akin to CAD, diabetes and hypertension, there is a higher incidence of NS amongst South Asians [1, 15]. This ethnic predisposition towards adverse outcomes, high burden of NS in our population, lack of reliable long term risk estimates for adverse cardiovascular outcomes in children with chronic renal conditions coupled with observations of insulin resistant dyslipidemic state which persists beyond remission, definitely merit further investigation of potential atherogenicity and cardiovascular comorbidity associated with childhood NS amongst Indians.

The primary objective of this study is to define extent of endothelial dysfunction by noninvasive evaluation for impairment of macrovascular and microvascular endothelial function in children with NS versus healthy controls, by assessment of brachial artery Flow mediated dilation (FMD) and cutaneous post-occlusive reactive hyperemia (PORH), respectively. Secondary objectives includes (a) To prospectively evaluate the temporal course of progression of endothelial dysfunction over 24 months of follow-up of these children in the macrovascular and microvascular endothelium as defined by repeating brachial artery FMD and cutaneous PORH (b) To evaluate in these children the association between endothelial dysfunction and morphological markers of subclinical cardiovascular comorbidity, defined by measurement of carotid intima media thickness (cIMT) and abnormalities on Nail Fold Capillaroscopy (NFC) and echocardiography (c) To look for numerical abnormality of circulating angiogenic cells and micro-particles as a marker of endothelial dysfunction in children with NS versus healthy controls, and (d) To assess biochemical marker of endothelial dysfunction in children with NS versus healthy controls.

## Methods/design

### Participating Centres

Army Hospital Research & Referral (AHRR), Delhi Cantt-10 and All India Institute of Medical Sciences (AIIMS), New Delhi.

### Ethical considerations

Study Protocol has been approved from Local Institutional Ethics Committee of AHRR and AIIMS participating centre.

### Design of study

Prospective cohort study.

## Inclusion and exclusion criteria

### Phase i: Case recruitment & baseline assessment of biomarkers of endothelial dysfunction

#### Cohort a: Children with Nephrotic syndrome (NS)

Consecutive cases of NS fulfilling the following criteria will be enrolled from the Pediatric Nephrology OPD of the participating institutions.

##### Inclusion

a) Age 1 and < 18 years age b) Estimated GFR > 60 ml/min/1.73 m2 c) Willingness to follow-up for subsequent 24 months.

##### Exclusion

a) Family history of premature atherosclerosis/stroke or familial hyperlipidemia b) Acute kidney dysfunction/injury at the time of recruitment c) Statin therapy at the time of recruitment d) Active systemic vasculitis e) Coexisting primary cardiovascular anomalies f) Anomalies of the limbs preventing diagnostic procedures g) Children with serious bacterial infection or thromboembolic episode will be recruited only after 12 weeks from the acute episode.

#### Cohort B: Healthy controls

1. Healthy siblings of the recruited children with NS between 1 and 18 years age. Children will be deemed healthy only if normotensive, with eGFR > 60 ml/min/1.73 m2 and with no systemic illness or serious bacterial illness in the preceding 12 weeks.
2. A detailed phenotyping of the disease profile and exposure to cardiovascular risk factors will be done for each child using a combination of clinical and laboratory examination. In addition a detailed nutritional assessment including dietary recall will be done in each child. Routine case specific laboratory assessments for clinical management of an individual case will continue as per standard treatment guidelines of Indian Society of Pediatric Nephrology [16, 17].
3. Baseline assessment of functional aberrations in microvascular and macrovascular endothelium will be done using LDF for cutaneous PORH and B-mode ultrasound for brachial artery FMD respectively.
4. Baseline screening for cardiovascular comorbidity will be done using 2D-echocardiography, high-resolution ultrasonography of carotid arteries and NFC.
5. In case of child being in relapse at the time of recruitment, a repeat assessment will occur at 12 weeks after the baseline assessment. Comparison of FMD and PORH between relapse and remission in same child will assess any short term effect of disease activity on these parameters.

### Phase ii: Prospective follow up and cardiovascular outcomes assessment

1. Children will be followed up to monitor the clinical course and undergo management of Nephrotic syndrome as per existing standard consensus guidelines [16, 17].
2. Noninvasive assessment of biomarkers of endothelial dysfunction and subclinical cardiovascular comorbidities along with biochemical estimates of cardiovascular risk will be carried out at defined time points.
3. At the end of 24 months from recruitment for a given case, final reclassification of disease phenotype based on the temporal profile of relapses/remission and steroid sensitivity will be done to categorize the subjects into the following categories as per standard definitions of Indian Society of Pediatric Nephrology [16, 17]: a. Steroid sensitive nephrotic syndrome (SSNS) b. Steroid resistant nephrotic syndrome (SRNS) c. Healthy controls
4. Venous blood plasma (in tubes containing EDTA (1.8 mg/ml) and citrated vials after centrifugation at 1500 rpm for 30 min at 4 degree Centigrade) and urine samples will be collected at baseline, 12 and 24 months and stored at − 80 C for batch assay of biochemical markers of endothelial dysfunction at a later date.

### Consent and recruitment

Witnessed written informed consent will be requested from all eligible patients or their next of kin (in case of patients with lower age group). Literate subjects or their next of kin will be requested to give their written consent by one of the physician investigators with a research staff witnessing the consent. Subjects or their next of kin who are not literate will have the contents of the consent form read out and explained by the investigator and their left thumb impression will be taken according to the current practice with a nurse as a witness. All questions from the subjects or their next of kin will be answered by the investigator. Potentially eligible patients will be recruited from the outpatient department and ward of the participating centre. Written informed assent will be obtained for children > 12 years age apart from oral assent for all children > 7 years of age in the presence of parents/legally authorised representatives.

### Baseline assessment

Standardized case record forms will be used to record patient history, general and NS examination. Clinical, laboratory and radiological findings are to be recorded.

### Protocols for noninvasive assessments

#### General protocols

- All investigations will be carried out in a minimum 4 h fasting state with parents instructed to refrain from giving any caffeinated drinks or chocolates in preceding 24 h as per standard guidelines [18–21]. Child will be acclimatized to a quiet room with comfortable temperature (22–25 °C) for at least 20 min prior to the testing in presence of the parents with informed consent. Timing of testing will be same time of the day for each child.
- The individual carrying out the investigation will be blinded to the clinical status of the child.
- At the time of study only image/data acquisition will occur. All images and electronic raw data will be captured, anonymized and stored in an electronic imaging repository established for this purpose for an offline analysis in a blinded manner. Image analysis and laboratory investigations will occur in a blinded manner by an investigator blinded to the clinical status of the child.
- For each investigation methodology, quality controls will be maintained by noting the inter-observer and intra-observer variability. It will be periodically assessed by rescanning of a random sample of 10 children with NS and 10 healthy controls. Coefficients of variability, intra-class correlation coefficients, and Bland Altman plots will be used for assessing variability.
- All invasive blood sampling will follow the noninvasive testing.

#### Specific protocols

A. Endothelial Dysfunction [18–21]

I. Microvascular Endothelial Function Equipment and method

Laser Doppler Flowmetry (Moor LDI 2 SIM; Moor Instruments Ltd., Axminster, UK) for quantification of post-occlusive reactive hyperaemia (PORH) which refers to the increase in skin blood flow following a brief arterial occlusion.

##### Technique

Brachial artery will be occluded using a pressure cuff placed around the upper arm and inflated up to 250 mmHg above the systolic blood pressure for 5 min followed by a quick deflation. Blood perfusion will be measured in Perfusion Units (PU) using a patented stepwise movement of the laser beam across the tissue to minimize background noise with a penetration depth of around 1 mm.

##### Endpoint

The primary endpoint will be the peak hyperaemia after the cuff release over the skin of volar aspect of forearm. In addition, area under the hyperaemic curve (AUCh), the raw value of the peak minus biological zero, increase in post-ischemic flow using area under the curve at baseline and post-ischemia will also be measured.

II. Macrovascular Endothelial Function

##### Equipment and method

High-resolution ultrasonography with 2D, color, and spectral Doppler imaging with high-frequency linear-array transducer (7 to 12 MHz) and ECG gating and digital recording, an automatically deflating pneumatic blood pressure cuff and stereotactic probe-holding device. Determination of FMD of the brachial artery following release of a 250 mmHg cuff occlusion for 5 min. The post-occlusive hyperemia leads to a shear stress over endothelium and consequent nitric oxide release and dilatation.

##### Technique

Child will rest supine with arm extended in a comfortable position. Blood pressure cuff will be placed below the antecubital fossa. Optimal resting 2D longitudinal image of the brachial artery will be obtained 2 cm above the antecubital fossa and Doppler signal will be recorded with the transducer perpendicular to the vessel wall to get a double line appearance. Cuff will be inflated to 250 mmHg for 5 min followed by rapid cuff deflation. Cine 2D and Doppler images will be recorded immediately after deflation and at every 60s after deflation till a maximum of 10 min after deflation to obtain the maximum lumen diameter after cuff release. Analysis of images will be done blinded in an offline fashion with careful attention to image quality after selecting images with a clear “double-line” sign from the same time during the cardiac cycle.. Intima-lumen interfaces will be used to measure the diameter by tracing the wall. Doppler flow velocity will be measured at baseline and at each point after deflation.

B. Subclinical Cardiovascular Comorbidity Assessments

1. Macrovascular

I. Estimation Of Carotid Intima Media Thickness (cIMT) Equipment

High resolution ultrasound scanner as for the FMD assessment with an optimal focus depth of 30–40 mm and frame rates greater than 15 Hz and automated edge detection algorithm for image processing with a 10 MHz linear array probe and a 128-radiofrequency line multi-array echo-tracking system for Quality Intima Media Thickness (RFQIMT) [Esaote BV, Maastricht, the Netherlands].

##### Method

The normal arterial wall is composed by two acoustic impedance interfaces: the transition between blood and intima, and the transition between media and adventitia. The distance between those two acoustic interfaces is the definition for IMT. Thus the cIMT will be defined by determining the thickness between the lines of Pignoli on B-mode ultrasound; with the first echogenic line representing the lumen intima interface, and the second line representing the media-adventitia interface. In addition, a high precision determination of the IMT on B mode Ultrasound with RFQIMT echo tracking will be the reference standard technique for IMT determination [This method has a minimum resolution of 21 μm with ability to measure change in diameters with 1.7-μm spatial resolution as against pure video imaging with a spatial resolution of 150 μm [22].

##### Technique

Image acquisition will be done as per standard Mannheim consensus protocol [23–25]. Briefly the child will be resting comfortably in the supine position with head turned 45° toward the side opposite the side being examined and patient’s carotid artery perpendicular to the plane of sound as much as possible. Farwall cIMT from 3 segments (distal common carotid 10 mm proximal to the carotid bulb, carotid bulb, and proximal internal carotid) will be measured at end diastole (R wave on ECG) and measurements on each side averaged to give the mean IMT for the right and left carotid arteries separately. In addition, carotid stiffness will also be assessed by using the B-mode image to place M-mode cursor across the common carotid artery 1 cm proximal to the bulb. Maximal and minimal lumen diameters from the right and left common carotid from M-mode echocardiography will be used in calculation of carotid stiffness.

##### Endpoint

The IMT from both sides will be further averaged to give the overall mean IMT. Any carotid plaque will be defined as a focal structure encroaching into the arterial lumen of at least 0.5 mm or 50% of surrounding IMT value, or an IMT value of > 1.5 mm.

##### Quality control

30 patients (15 nephrotic and 15 controls) will be revaluated and their IMT reassessed by a second ultrasonographer, in order to assess inter- and intra-observer variability to have an acceptable variation < 9%.

2. Microvascular

II. Nailfold Video capillaromicroscopy Equipment & Method

Nailfold videocapillaroscope with a minimum 300X magnification with digital output [26].

##### Technique

Middle 3 mm of nailfold of ring finger of non-dominant hand will be examined using oil drop magnification.

##### Endpoint

Capillary density: Number of loops per mm Capillary dimension: The first 5 capillaries from the right hand edge of the nailfold in which arterial, apex, and venous widths could all be discerned would be chosen. Apex, arterial, venous, and total capillary width (the width at the widest point) of each capillary will be measured. Mean across 5 loops will be calculated for each of apex, arterial, venous, and total capillary widths. All loop dimensions will be log transformed to achieve normality. Architectural pattern: 6 descriptive classes: cuticilis, open, tortuous, crossed, bushy, and bizarre will be noted. Intra- and inter-observer variability/reliability of < 10% will be acceptable [27].

III. Echocardiography Assessment Equipment & Method

Standard transthoracic echocardiography using 2.5–7MHZ probes equipped with tissue Doppler imaging technology as per published studies [8, 28, 29].

##### Technique

Evaluation by cardiologist blinded to the status of the subjects. All patients will undergo echocardiographic examination by a well-experienced pediatric cardiologist blinded to the diagnosis in order to reduce bias. Two dimensional images will be obtained using standard views in the left lateral decubitus position. Images will be acquired at passive end-expiration to minimize global cardiac movement from standard parasternal long axis and apical planes. Left ventricle (LV) dimensions will be obtained in the standard views. LV end systolic and end diastolic volumes will be calculated by using the modified Simpson’s method, and ejection fraction will be calculated from the LV end systole and end diastolic volumes. The left ventricular mass index (LVMI) (g/m2) will be calculated using the Devereux’s formula by the following equation: Left Ventricular Mass (LVM) = 0.80 [1.04 × (interventricular septal thickness + posterior wall thickness + end-diastolic diameter) 3 - (end-diastolic diameter) 3] + 0.6. The LVMI will be calculated as LVM divided by the body surface area (BSA). LVMI is calculated according to end-diastolic measurements of LV posterior and septal wall thickness and internal dimension, standardizing all to body surface area. Circumferential cardiac contractility and shortening will also be measured. LVMI > 38 g/Hg2.7 will be defined as LVH.

##### Quality control

Re-evaluation of 30 patients re-assessing LVMI by a second pediatric cardiologist, in order to assess inter- and intra-observer variability.

IV. Flow Cytometry for Circulating Angiogenic Cells and Derivatives [30]

##### Methodology

15 ml of venous blood will be drawn into citrated and EDTA vials. The initial 5 ml will be used for biochemical testing. The subsequent 10 ml will be used for flow cytometry. Initial 3 mL of blood will not be used to avoid contamination with endothelial cells from the puncture wound of the vein. After centrifugation at 1500 rpm for 30 min at 4C, the samples will be processed further as follows.

- Assay of Circulating CD31+/Annexin V+ Apoptotic Microparticles

Plasma derived from 10 mL citrate-buffered blood will be immediately centrifuged at 13,000 g for 2 min to generate platelet poor plasma. Fifty microliters of platelet-poor plasma will be incubated with 4 mL of phycoerythrin (PE)-conjugated monoclonal antibody against CD31 followed by incubation with fluorescein isothiocyanate (FITC)-conjugated annexin V according to the manufacturer’s instructions. IgG FITC (Pharmingen, San Jose, California, USA) will serve as the negative control. Fluorescence-activated cell sorting (FACS) analysis will be performed immediately after staining using a FACS Calibur flow cytometer. Standard beads (Nile Red fluorescent particles, 1.7–2.2 μm, catalog number 556261, BD; Megamix 0.5–3 μm (Biocytex, France) will be used to identify microparticles in gating cells by FACS so as to standardize the set-up of MP analysis region (0.5–1 μm) and guarantee the stability of the settings. CD31+/annexin V+ microparticles will be defined as particles positively labeled for CD31 and annexin V. (CD31+/annexin V+). Data will be analyzed using Cell quest software

b) Assay of Circulating Angiogenic Cells

A volume of 1000 microL peripheral blood will be incubated for 30 min in the dark with monoclonal antibodies against human kinase insert domain-conjugating receptor (KDR) followed by PE-conjugated secondary antibody, with the fluorescein isothiocyanate (FITC)-labeled monoclonal antibodies against human CD45, and PE-conjugated monoclonal antibody against human CD133 and with FITC-conjugated or PE-conjugated monoclonal antibodies against human CD34 and KDR. Isotype-identical antibodies will serve as controls. After incubation, cells will be lysed, washed with phosphate-buffered saline (PBS), and fixed in 2% paraformaldehyde before analysis. Each analysis will include 100,000 events. Numbers of circulating EPCs will be gated with monocytes and defined as CD34 + KDR + CD133+. For CECs, CD146 will be used.

### Sample size calculation

Sample size calculation was based upon the reported differences in mean brachial artery FMD and cIMTs in children below 18 years of age in published literature [18] as well as our previous unpublished study in 52 children with NS and 50 healthy controls which looked at cIMT and FMD as the primary outcomes [19]. FMD and cIMT are the most validated indices of endothelial dysfunction and subclinical atherosclerosis in published literature [18]. Data on LDF for PORH in children remains limited. Reported values of mean (± SD) change from baseline of FMD in normal children between 9 and 16 years have ranged between 4.73 ± 4.38% to 7.7 ± 4.0% while the reported maximum mean cIMT in normal children below 18 years of age has been 0.50 + − 0.05 mm3.

A. For Cross Sectional Comparisons during Phase I and II

Assuming a conservative estimate of minimum difference of FMD of 3% between normal and nephrotic syndrome with a standard deviation (SD) of 5%, a minimum of 46 cases each of NS and healthy controls will be required for a study with power of 80% at a two-sided 0.05 significance level. This sample size will be able to detect a difference of 0.03 mm of cIMT between children with NS and healthy controls with a power of 80% at a 0.05 significance level. With a 5% attrition rate due to mortality or loss to follow up for the entire study, a minimum sample size of 50 children with NS and 50 healthy children is proposed during phase I of the study. Any subsequent cases of incident/prevalent nephrotic syndrome presenting to the OPD after the 12 months recruitment phase gets over will also be added onto the repository for future analysis. Hence at end of 36 months of study, a minimum of 70 cases each of SSNS, SRNS and healthy controls will be required after final classification of phenotype of NS for a post-hoc Bonferroni adjustment for comparison of 3 means (2 groups of SSNS and SRNS compared to a control healthy group) of FMD and cIMT. Between the 2 participating institutions this number is expected to be available by 36 months of study.

B. For Prospective Follow-Up during Phase II

For the phase 2 of study with prospective follow up for progression of endothelial dysfunction towards subclinical atherosclerosis as determined by cIMT as the gold standard, if total of 95 children (NS and controls) were to be followed for 24 months after an accrual period of 12 months, assuming a median time of 12 months for a detectable difference of cIMT of > 0.004 mm over 12 months between children with NS and healthy controls, the probability is 80% that the study will detect a difference at a two sided 5.0% significance level, if the true hazard ratio is 2.0. With these preceding assumptions again minimum of 50 children with NS and 50 healthy controls will need to be recruited and followed up over 24 months.

### Statistical analysis

Descriptive statistics will be used to characterize the entire population and subgroups of interest at baseline and follow-up. Summary statistics such as means, medians, standard deviations and ranges will be used to describe continuous variables, and frequencies will be tabulated for categorical variables. For outcomes collected longitudinally, Kaplan Meier survival curves, scatter plots and grouped box plots will be used to examine assumptions of survival free of adverse outcome or relapses, and linearity and symmetry of data. Repeated measures analysis will be applied for data derived at multiple time points for same child to study correlation with extent and duration of proteinuria. Cox regression analysis will be applied to analyze the time to event data of the primary or secondary endpoints collected in both cohorts of NS and healthy children and assess association of the outcome hazard with clinical covariates of interest. Correlation of markers of endothelial dysfunction and cIMT will be done with extent and duration of proteinuria as defined by the status of remission and number of relapses per year. cIMT will be used as the gold standard for subclinical vasculopathy for evaluation of association with progression of endothelial function. IMT is chosen for diagnostic evaluation study for other biomarkers of cardiovasculopathy since it is the most well characterized surrogate marker of subclinical atherosclerosis. *P*-value < 0.05 will be considered as significant. Statistical analysis will be performed using SPSS Version 17.0.

### Strategies to minimize losses to follow-up

All the patients will be followed up at 12 and 24 months after the baseline assessments. To minimize the risk of retrieval bias, two to five telephone numbers (both mobile and landline) along with their army service number will be noted from the patients or his/her relatives at the time of recruitment. A trained research worker will attempt to contact the participants one month, one week and two days prior to their respective follow up.

### Outcomes and their measurement

The expected outcomes of the CVONS study will able to assess extent of impairment in macrovascular & microvascular endothelial function due to childhood NS in the absence of usual confounders in adults like smoking, alcohol, coronary heart disease, diabetes. This study may able to delineate progression of endothelial dysfunction in relation to - disease activity (extent and duration of proteinuria) and structural markers of subclinical atherosclerosis (cIMT). It may provide preliminary normative data for Indian children for vascular measurements. It may also provide correlation of cardiovascular risk factors with endothelial dysfunction and subclinical atherosclerosis for pediatric cardiovascular risk stratification in nephrotic syndrome.

## Discussion

This study aims to provide a better understanding of the epidemiology of endothelial dysfunction and associated subclinical cardiovascular co-morbidity in childhood NS. We hypothesize functional as well as structural alterations in the entire microvascular, macrovascular and cardiac endothelium due to NS and seek to evaluate the same using a combination of noni-nvasive imaging, biochemical and flow cytometry based biomarkers. Amongst the imaging biomarkers, our study may encounter certain challenges during the use of LDF, RF-IMT and FMD. During the procedure of LDF, subjects are restricted to immobilized to get the accurate cutaneous perfusion flux. It is notable that LDF does not provide a direct measurement of blood flow, but rather is an index of cutaneous perfusion [31, 32]. As yet there is no preliminary normative data on flux values as well as flux variation in proximal and distal vascular beds of forearm and we seek to obtain them from the controls. Seasonal temperature variation causing deviation in the flux values is obviated by measurements at uniform ambient temperature after a rest period. Short neck length in younger children in relation to the vascular probe makes assessment of the internal carotid artery (ICA) thickness difficult especially since the ICA tends to dip inwards in them [22, 23]. Repetitive swallowing prolongs the RFQIMT acquisition times. Carotid bifurcation and the inferior thyroid artery will form reference points for manual measurement of IMT. Data for objective assessment of nail fold capillaroscopy for non rheumatological disorders in childhood too remains sparse [25, 26].

FMD assessment is highly observer dependent and, variations in cuff placement/positioning have to be minimized. Visualization of brachial artery in young children can be difficult and there is a need to ensure measurement at same point in cardiac cycle. M-mode ultrasonography offers high spatial and temporal resolution and is therefore suitable for diameter measurement of pulsatile vessels. Till yet there is no any normative data for age related cut-off values for the diameter of brachial artery. Depending upon pulse pressure and vascular stiffness, arterial diameter may vary quite considerably across a single cardiac cycle. In some subjects, the change in diameter may be as much as one millimeter, which, if unaccounted for, may completely confound the assessment of FMD [33–35]. In our study we seek minute-wise sequential data pertaining to the flow mediated brachial artery parameters. Further FMD values obtained by M-mode and by the conventional B-mode ultrasonography on longitudinal images will be compared. M-mode brachial artery diastolic and systolic diameter measurement is feasible, suitable, and accurate for the assessment of FMD even without the need for electrocardiography.

Flow cytometry evaluation of endothelial progenitor cells, circulating endothelial cells and endothelial micro-particles is a challenge and pediatric data is lacking [11]. Scattered within the vascular wall, these cells participate in angiogenesis and vasculogenesis and support regeneration of epithelial cells and during the course of chronic cardiovascular and kidney disease they undergo premature senescence and fragmentation to generate endothelial micro-particles. No study till date has tried to correlate these novel cellular markers with imaging biomarkers of endothelial dysfunction.

Overall CVONS study will able to prospectively assess the extent of functional and structural impairment in macrovascular & microvascular endothelial function and the corresponding biochemical and cytological correlates due to childhood NS in the absence of usual confounders in adults like smoking, alcohol, coronary heart disease, diabetes. In the prospective phase, delineation of progression of endothelial dysfunction in relation to disease activity (extent and duration of proteinuria) and structural markers of subclinical atherosclerosis (cIMT) will be focused upon. Preliminary prospective normative data for Indian children for vascular measurements is sought to be generated. Better understanding of the epidemiology of endothelial dysfunction and associated subclinical cardiovascular co-morbidity in childhood NS is expected. Findings on characterization of prevalence of endothelial dysfunction and subclinical markers may be used to design future randomized controlled trials for evaluating the efficacy of preventive and therapeutic interventions in reducing the incidence of cardiovascular disease.

## Abbreviations

CAD: Coronary Artery Disease
CD: Cluster of Differentiation
CEC: Circulating Endothelial Cells
cIMT: Carotid Intima Media Thickness (cIMT)
CKD: Chronic Kidney Disease
CVONS: Cardiovascular Outcome in Nephrotic Syndrome
EPC: Endothelial Progenitor Cells
FACS: Fluorescence-Activated Cell Sorting
FITC: Fluorescein Isothiocyanate
FMD: Flow Mediated Dilation
IMT: Intima Media Thickness
KDR: Kinase insert Domain-conjugating Receptor
LDF: Laser Doppler Flowmetry
LDI: Laser Doppler Imaging
LV: Left Ventricle
LVMI: Left Ventricular Mass Index
NFC: Nail Fold Capillaroscopy
NO: Nitric Oxide
NOS: Nitric Oxide Synthethases
NS: Nephrotic Syndrome
PORH: Post Occlusive Reactive Hyperaemia
RF: Radiofrequency
SRNS: Steroid Resistant Nephrotic Syndrome
SSNS: Steroid Sensitive Nephrotic Syndrome
